# The bee tree of life: a supermatrix approach to apoid phylogeny and biogeography

**DOI:** 10.1186/1471-2148-13-138

**Published:** 2013-07-03

**Authors:** Shannon M Hedtke, Sébastien Patiny, Bryan N Danforth

**Affiliations:** 1 Department of Entomology, Cornell University, Ithaca, NY, USA; 2 Laboratory of Zoology, Mons University, Mons, Belgium

## Abstract

**Background:**

Bees are the primary pollinators of angiosperms throughout the world. There are more than 16,000 described species, with broad variation in life history traits such as nesting habitat, diet, and social behavior. Despite their importance as pollinators, the evolution of bee biodiversity is understudied: relationships among the seven families of bees remain controversial, and no empirical global-level reconstruction of historical biogeography has been attempted. Morphological studies have generally suggested that the phylogeny of bees is rooted near the family Colletidae, whereas many molecular studies have suggested a root node near (or within) Melittidae. Previous molecular studies have focused on a relatively small sample of taxa (~150 species) and genes (seven at most). Public databases contain an enormous amount of DNA sequence data that has not been comprehensively analysed in the context of bee evolution.

**Results:**

We downloaded, aligned, concatenated, and analysed all available protein-coding nuclear gene DNA sequence data in GenBank as of October, 2011. Our matrix consists of 20 genes, with over 17,000 aligned nucleotide sites, for over 1,300 bee and apoid wasp species, representing over two-thirds of bee genera. Whereas the matrix is large in terms of number of genes and taxa, there is a significant amount of missing data: only ~15% of the matrix is populated with data. The placement of the root as well as relationships between Andrenidae and other bee families remain ambiguous, as several alternative maximum-likelihood estimates fall within the statistically credible set. However, we recover strong bootstrap support for relationships among many families and for their monophyly. Ancestral geographic range reconstruction suggests a likely origin of bees in the southern hemisphere, with Melittidae ancestrally located within Africa, and Halictidae, Colletidae, and Apidae within the New World.

**Conclusions:**

Our study affirms the monophyly of each bee family, sister-taxa relationships between Apidae and Megachilidae (the ‘long-tongued bees’), between Colletidae and Stenotritidae, and between Colletidae + Stenotritidae and Halictidae. Our analyses reject a Colletidae-basal hypothesis for family-level relationships and instead support Melittidae as sister to the remaining bees. Southern hemisphere vicariance likely played an important role in early diversification within many bee families.

## Background

Bees (Hymenoptera: Apoidea: Anthophila) provide a rich system for exploring the evolutionary consequences of a wide variety of life history characteristics. Bees provision larvae with pollen and nectar, a trait that evolved in the early- to mid-Cretaceous from carnivorous, wasp ancestors [1-5]. It has been suggested that this transition from carnivory to pollenivory led to rapid diversification and expansion of bee lineages as a result of the exploitation of pollen as a novel resource (i.e., a key innovation [6,7]). Within bees, a number of life history traits have evolved multiple times both within and among bee families, including diet specialization, eusociality, and social parasitism (reviewed in [8]). A robust phylogeny is fundamental to determine how changes in these life history traits have affected behavior, geographic range, phenology, susceptibility to habitat loss or pathogens, and gene or genome evolution.

**Figure 1:**
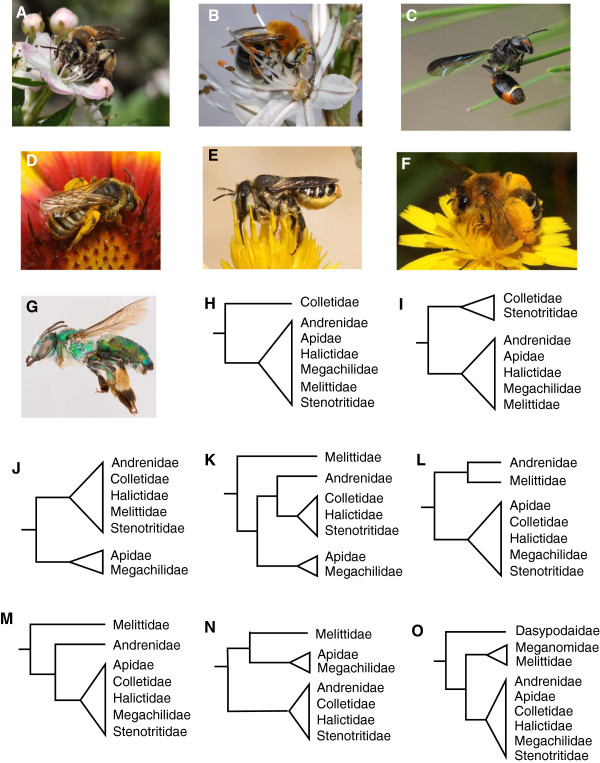
**Previously published hypotheses for relationships among bee families. A**. *Andrena crataegi* (Andrenidae) [photo credit: Phil Huntley-Franck], **B**. *Habropoda tarsata* (Apidae) [photo credit: Jelle Devalez], **C**. *Hylaeoides concinna* (Colletidae) [photo credit: Kristi Ellingsen], **D**. *Halictus* sp. (Halictidae) [photo credit: Nicolas Vereecken], **E**. *Lithurgus chrysurus* (Megachilidae) [photo credit: Nicolas Vereecken], **F**. *Dasypoda hirtipes* (Melittidae) [photo credit: Nicolas Vereecken], **G**. *Ctenocolletes smaragdinus* (Stenotritidae) [photo credit: Laurence Packer, York University: Bee Tribes of the World photographic project]. **H-O**. Alternative topologies for relationships among bee families, including the placement of the root.

Over 60 molecular phylogenies of bees have been published to date, and yet phylogenetic relationships among the seven families of bees remain highly controversial, with conflicting results obtained among and even within studies (reviewed in [8]). Morphological analyses have placed the plasterer or cellophane bees, family Colletidae (Figure 1C), as sister to the remainder of the bee families (Figure 1H), or basal together with Stenotritidae (Figure 1I), a small family with limited Australian distribution (Figure 1G). This result is largely driven by a single morphological characteristic shared by apoid wasps and colletid bees: a bilobed (or bifid) tongue or glossa [9]. Subsequent molecular and morphological analyses have not supported a Colletidae-basal hypothesis, and the bilobed glossa may be an independently-derived character associated with the application of the cellophane-like lining to cell and burrow walls [10-13].

The families Megachilidae (including leaf-cutter bees, carder bees, mason bees, and others; Figure 1E) and the family Apidae (including honey bees, bumble bees, orchid bees, and others; Figure 1B) clearly form a monophyletic group (the “long-tongued” bees), supported by the shared possession of highly modified first and second labial palpal segments [3]. The remaining bee families (Andrenidae [Figure 1A], Colletidae, Halictidae [Figure 1D], Melittidae [Figure 1F], and Stenotritidae) form the loosely-defined “short-tongued” bees. Relationships among short-tongued bees are unclear. Monophyly of short-tongued bees has not been supported by most previous morphological or molecular studies, although one analysis of three nuclear genes supported a tree in which short-tongued and long-tongued bees are reciprocally monophyletic (Figure 1J; [14]). The family Andrenidae has been suggested to be sister to a clade containing Colletidae, Stenotritidae, and Halictidae (Figure 1K; [12,14,15]), sister to Melittidae (Figure 1L; [16]), or sister to all bees except Melittidae (Figure 1M; [16]).

Molecular studies have proposed Melittidae as monophyletic and sister to the remainder of the bees (Figure 1K, M; [16,17]) or sister to the long-tongued bees (Figure 1N; [18]). Both morphological and molecular [9,12,15,17] studies have supported a tree in which Melittidae is a paraphyletic group at the base of bee phylogeny (Figure 1O). Such a topology would lend support to elevating the three melittid subfamilies (Dasypodainae, Melittinae, and Meganomiinae) to families (as suggested by [9]).

An obvious strategy to improve our ability to distinguish among alternative hypotheses is to increase both taxonomic sampling and the number of genes sampled for phylogenetic analysis ([19-24]). Increased taxon sampling can improve statistical support for accurate phylogenetic estimates even when the taxa added have incomplete information [25-27]. We estimate phylogenetic relationships among an unprecedented number of apoid taxa by combining publically-available data from multiple, independent sources. We confined our analyses to DNA sequences of nuclear protein-coding genes, which have more power than mitochondrial genes in recovering older relationships [28-30] and are considerably more straight-forward to align compared to ribosomal genes. We tested alternative phylogenetic relationships from the literature for statistical significance. Finally, we provide the first global biogeographic analysis to explore bee historical biogeography at the level of family and subfamily.

## Results and discussion

### The bee tree of life

**Figure 2:**
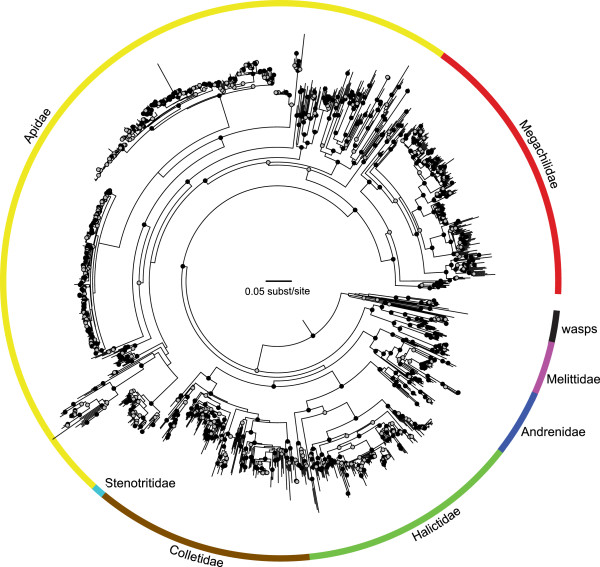
**Maximum-likelihood estimate of relationships among bee species.** Circles at nodes represent bootstrap proportions after 100 nonparametric bootstrap replicates: black >=0.8, grey >=0.5. Nodes with bootstrap proportions less than 0.5 are without circles. Tips are unlabelled to reduce visual complexity; the labelled species tree is available in Newick format in Additional file 1.

We have assembled the largest molecular data set for analyzing higher-level (family, subfamily, tribal) relationships among bees to date. Our data set includes 349 of the approximately 500 currently recognized bee genera [31], including over 17,000 sites concatenated from DNA sequences of twenty nuclear protein-coding genes (Table 1). Although the alignment contains a substantial amount of missing data (~85%), we returned a phylogeny with high bootstrap proportions for the monophyly of each bee family (Figure 2, Table 2). We obtained strong bootstrap support for several additional clades: the long-tongued bees (Apidae + Megachilidae), Colletidae + Stenotritidae, and a clade containing Halictidae + Colletidae + Stenotritidae. Melittidae is weakly supported as sister to all other bees, consistent with the hypothesis that the root of bee phylogeny falls near Melittidae rather than Colletidae (Table 2). Andrenidae is sister to Colletidae + Stenotritidae + Halictidae (Figure 1K), but with a bootstrap proportion of only 0.47 (Table 2, Additional file 1).

**Table 1:** Number of species sampled per gene

**Taxon**	**abdA**	**AK**	**bub3**	**cad**	**camkii**	**dnk**	**ecrb1**	**ef1af1**	**ef1af2**	**fem**	**gk**	**nak**	**or2**	**pepck**	**rho**	**polII**	**usp**	**vas**	**white**	**wg**
**Apoid wasps**																				
Sphecidae	1			1				2	8			3			4	3				11
Crabronidae				10				3	16			13			11	12				22
Ampulicidae									1											1
Total apoid wasps	1			11				5	25			11			15	15				34
**Anthophila (bees)**																				
Andrenidae				12				11	50			43			43	48				10
Apidae	2	443	24	35	12	18	4	79	613	5	21	157	6	259	547	223	4	5	12	157
Colletidae		17		8				8	166			19			126	29				171
Halictidae				13				17	157			18			163	29				126
Megachilidae				206				5	209			109			209	30				24
Melittidae				21				9	40			36			42	41				9
Stenotritidae				1				1	4			2			1	2				4
Total Anthophila	2	460	24	296	12	18	4	130	1273	5	21	348	6	259	1149	408	4	5	12	501

**Table 2:** Effects of subsampling on bootstrap proportions for select clades

**Hypothesis**	**20 genes (species)**	**20 genes (genera)**	**10 genes (species)**	**7 genes (species)**
Andrenidae monophyletic	0.99	1	0.99	1
Apidae monophyletic	0.86	1	0.97	1
Colletidae monophyletic	0.98	0.999	0.99	1
Halictidae monophyletic	0.9	1	1	1
Megachilidae monophyletic	1	1	1	1
Melittidae monophyletic	1	0.964	0.98	1
Stenotritidae monophyletic	1	1	1	1
Colletidae + Stenotritidae	0.98	1	1	1
Apidae + Megachilidae (long-tongued bees)	0.87	0.998	0.97	1
Halictidae + Colletidae + Stenotritidae	0.88	0.985	0.99	0.97
Andrenidae + Halictidae + Colletidae + Stenotritidae	0.47	0.578	0.52	0.59
Short-tongued bees monophyletic	0.37	0.108	0.24	0.36
Melittidae + long-tongued bees	0	0.16	0.03	0.04
Melittidae + Andrenidae	0.1	0.034	0.07	0.1
Melittidae (paraphyletic or monophyletic) basal	0.57	0.861	0.79	0.57
Melittidae monophyletic and basal	0.57	0.845	0.68	0.57
Dasypodainae basal	0	0.011	0.02	0
Melittidae, (Andrenidae,(other bees))	0.33	0.362	0.31	0.57
Colletidae basal	0	0	0	0
Colletidae + Stenotritidae basal	0	0	0	0

At the subfamily and tribal levels, our phylogeny is broadly congruent with molecular phylogenies published for particular families, and where it is not, bootstrap support values fall below 0.75. Within Apidae, discrepancies between this study and that of Cardinal et al. [32] include the placement of Anthophorini, Caenoprosopidini, Ammobatoidini, Manuelini, and Apini/Euglossini. Our phylogeny is consistent with their finding that Apinae is not monophyletic: we recovered a large clade composed of the majority of cleptoparasitic species of Apinae and Nomadinae. Our inferred relationships among megachilid tribes differs from Litman et al. [33] only in the placement of Lithurgini as basal to, rather than sister to, Pararhophitini. Our phylogeny lacks resolution within Colletidae, but all well-supported nodes are also found in the estimate of Almeida et al. [34]. Within Halictidae, Thrincostomini is sister to Halictini, in contrast to Danforth et al. [35], but our phylogeny is otherwise congruent with theirs. Finally, the only discrepancy between relationships recovered within Melittidae in this study and that of Michez et al. [17] is that we do not recover Dasypodaini as monophyletic.

### Hypothesis testing

Many of the family-level relationships in our tree are reasonably well-supported based on bootstrap proportions. Despite this, we could not reject five of our plausible alternatives to rooting the bee tree with high statistical support (Table 3). The placement of Colletidae as basal to the remainder of the bees and the monophyly of long-tongued bees + Melittidae can confidently be rejected as failing to fit our data, even after Bonferroni correction for multiple testing (p < 0.01). The remaining possible topologies all fall within the confidence set of both the approximately unbiased and weighted Shimodaira-Hasegawa tests. The BIC places only one hypothesis within this 95%, in agreement with the maximum-likelihood estimate (Figure 2).

**Table 3:** Statistical tests of alternative topologies

**Hypothesis**	**WSH**	**AU**	**BIC**
K*: Andrenidae + Halictidae + Colletidae + Stenotritidae	**0.990**	**0.841**	**1.0**
M: Melittidae, (Andrenidae, (other bees))	**0.677**	**0.453**	>0.001
J: Monophyly of short-tongued and long-tongued bees	**0.413**	**0.181**	>0.001
O: Dasypodainae basal	**0.447**	**0.215**	>0.001
I: Colletidae + Stenotritidae basal	**0.263**	**0.118**	>0.001
L: Melittidae + Andrenidae	**0.216**	**0.097**	>0.001
H: Colletidae basal	0.001	0.001	>0.001
N: Melittidae + long-tongued bees	0	>0.001	>0.001

Probabilities that a given topology falls within the 95% confidence set (reject after Bonferroni correction when p < 0.01) under the weighted Shimodaira-Hasegawa test (WSH), approximately unbiased test (AU), and Bayesian Information Criterion (BIC). Letters refer to the topologies in Figure 1. For each hypothesis, values that indicate the topology falls within the 95% confidence set are in bold. * = maximum-likelihood estimate.

Phylogenetic inference based on DNA sequence data can be affected by saturation—when phylogenetic signal among sequences is stochastically lost over time. Saturation occurs more rapidly in fast-evolving sites, such as the third codon position, and removal of these sites may increase phylogenetic accuracy [19]. We estimated phylogenetic relationships after excluding the third codon position from our DNA sequence alignment. The resulting maximum-likelihood estimate was unresolved, suggesting that third codon positions do contain phylogenetic signal (Additional file 1).

Several genes are relatively sparsely sampled across bee families (Table 1; Additional file 2), and phylogenetic analyses of individual genes returned poorly-supported topologies (Additional file 1). While even incomplete data are often informative in concatenated analyses [25-27], particularly when parameterizing the model of sequence evolution [26], missing sequences could instead decrease statistical support for particular nodes, or cause an increase in bootstrap support due to systematic error. Our data set was not phylogenetically decisive [36]: some taxonomic triplets in our concatenated data set were not sequenced for the same gene. Partial tree decisiveness based on 1,000 simulated, equiprobable trees [36] was relatively high (0.946). We examined the subtrees generated by pruning taxa from the maximum-likelihood estimate to match taxon sampling for each data partition. The number of trees that could be built from these observed subtrees, or the terrace size [37], is huge (~1 billion). When terrace size is high and phylogenetic decisiveness is inadequate, the pattern of taxonomic overlap among partitions may affect phylogenetic accuracy. However, the BUILD tree, which is an Adams consensus of trees in the terrace [38], did return the same family-level relationships as the maximum-likelihood estimate (Additional file 1). This suggests that incomplete taxonomic overlap across data partitions may be more problematic when examining species relationships within families.

**Figure 3:**
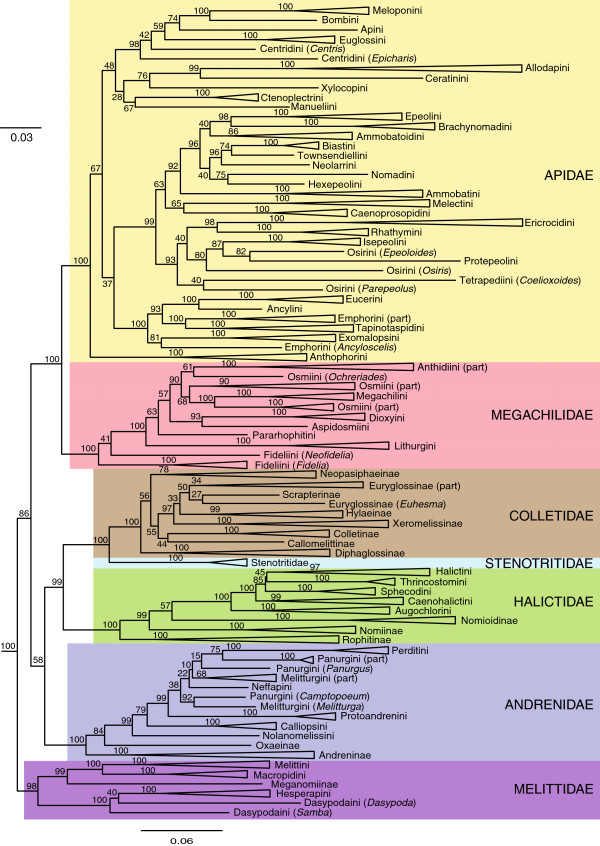
**Maximum-likelihood estimate of relationships among bees.** Tree estimated using one randomly-selected DNA sequence per gene per genus. Numbers at nodes represent bootstrap percentage after 100 replicates. To enhance visualization of relationships, wasp outgroups were removed, and clades of tribes and subfamilies containing more than one genus have been collapsed. The tree with all genera is in Additional file 3. When taxonomic groups are not monophyletic, clades are labelled with the taxonomic name and either “(part)” if more than one genus is included, or “(*Genus*)” if only one terminal taxon is in the taxonomic group.

We assessed the effects of reducing the proportion of missing data on our analysis by excluding poorly-sampled genes. When we concatenated only those genes sampled for at least two bee families (10 genes with 78.4% missing data, partial tree-wise decisiveness 0.979) or at least four families (7 genes with 71.7% missing, partial tree-wise decisiveness 0.981), the resulting maximum-likelihood estimates supported the same family-level relationships as for the complete data set (Table 2; Additional file 1). We also reduced the empty cells in our matrix by combining data within genera, such that each genus was represented by one randomly-selected species per gene (an average of 77% missing over 10 replicate alignments, partial tree-wise decisiveness 0.987). These maximum-likelihood estimates all returned either the same topology as the species-level tree, or a topology in which Andrenidae is sister to all bees except Melittidae (Additional file 3). When combining sequences at the genus-level, terrace size improved dramatically: only one tree could be returned from the taxon triplets observed across gene subtrees. We used one randomly-selected genus-level estimate (Figure 3) when examining historical biogeography of bees. The effects of these treatments do not change the overall conclusions: bee families are monophyletic, but uncertainty remains in the placement of Andrenidae relative to other bee families (Table 2).

Missing data also come in the form of missing taxa, and certain groups are less well-sampled than others. Andrenidae has a lower proportion of genera sampled relative to other bee families, and its phylogenetic placement is uncertain. Only about 47% of andrenid genera have more than one gene sequenced (and thus retained in our data set), compared to the average within families (excluding Stenotritidae, which has complete generic-level sampling) of 65% and the average across all bees of 79%. Increasing taxon sampling across partitions for this group may be necessary to resolve its relationship to other bees, as this additional sequence data would contain information about internal nodes.

Missing data, in terms of taxon and gene sampling, may not be the only source of weak statistical power when distinguishing among alternative hypotheses for relationships among bees. Branch lengths along the backbone of the tree are noticeably shorter than average. The branch leading to the melittid bees, the branch between Melittidae and the remainder of the bees, and the branch that determines the placement of Andrenidae are all within the lower one third of the branch length distribution. Thus, the uncertainty of early bee history appears to be due, in part, to short branches among families. This suggests that major lineage differentiation occurred within a relatively short amount of time early in bee history. Incomplete lineage sorting or hybridization between lineages early in bee history could also obscure bifurcations. This problem is not unique to bees: similar difficulties in resolving early branching patterns based on molecular sequence data have also plagued researchers working on butterflies [25], ants [39], and birds [40].

### Comments on the bioinformatics approach

Database mining is not without problems. First, our ability to objectively curate data is limited. The inclusion of *Ceratina japonica* within a clade of *Apis* spp. rather than with other *Ceratina* spp., or of the type species of Anthophorini, *Anthophora plumipes*, within the eucerine bees (Additional file 1), is suggestive of either incorrect species identification, DNA contamination, or error in uploading sequence to the GenBank database. Our skepticism that the placement of these species reflects evolutionary history is warranted. *Ceratina* are morphologically very distinct from *Apis*, even to a non-expert in field conditions, and error in species identification is highly unlikely. The longest ef1af2 sequence for *Ceratina japonica* (DQ149700), and thus the one selected by our bioinformatic pipeline, is identical to the DNA sequence in the *Apis mellifera* genome (NM001014993), while a shorter sequence (AY250212) is more similar to other *Ceratina* (i.e., best blast hit). A similar problem occurs for *Anthophora plumipes*, whose sequence for RNA polII (GU245385) is identical to that of *Eucera frater* (EU184737), although these species are otherwise genetically, morphologically, and geographically disparate. We did not prune these erroneously-identified sequences from our dataset prior to analysis, but when DNA sequences from one species are concatenated with those of another, this will introduce inaccuracy into phylogenetic reconstruction. Other unidentified taxonomic errors may be present within our dataset. One potential solution would be to ensure that a given sequence has as its best blast hit a member of the same species or genus prior to alignment, assuming that named genera represent monophyletic groups and that such data are available.

While not a source of phylogenetic error, several records refer to taxa that have subsequently been synonymized. For example, within Apidae, *Inquilina* is no longer recognized as valid, and has been synonymized with *Exoneura*; within Megachilidae *Fideliopsis* is now a subgenus of *Fidelia*. Since the NCBI taxonomic databases are not always up-to-date with the latest classifications, our bioinformatic pipeline treats these genera as separate entities*.* The solution would be to manually curate sequence records to reflect the current state of taxonomy (as in Figure 3).

### Historical biogeography

**Figure 4:**
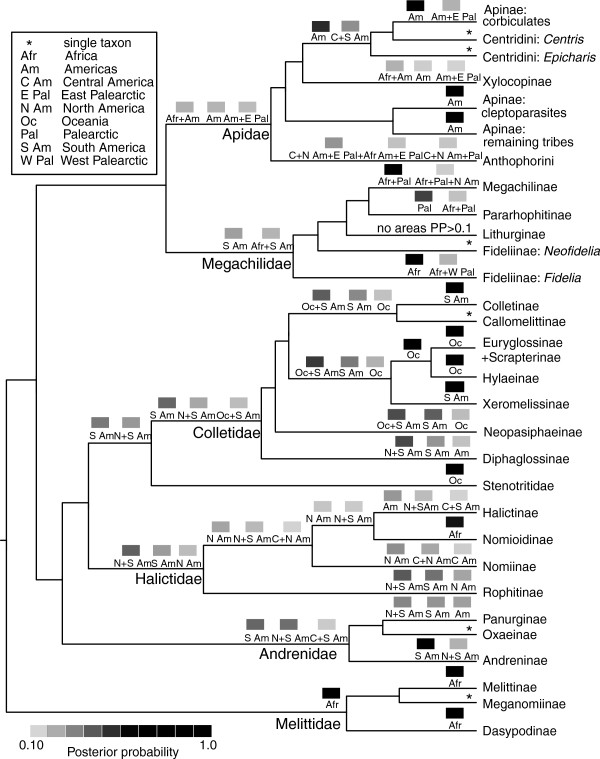
**Historical biogeography of bees.** Cladogram (based on maximum-likelihood estimate) indicating reconstructed ancestral ranges of bee genera. Only results for subfamilies are presented. Boxes at each node represent the the top 3 reconstructed ranges according to posterior probability; posterior probabilities less than 0.10 are not represented. Boxes are shaded by posterior probability.

In our biogeographic analyses (Figure 4, Additional file 4), the ancestral distributions of many groups of bees remain uncertain, especially at the family level and for groups with widespread distributions (e.g., Lithurginae). We could not clearly identify a sample bias in our data set that would drive this uncertainty. For example, we have identified the family Andrenidae as more poorly sampled than other groups in our phylogeny. However, we are primarily missing South American taxa in the andrenid tribes Calliopsini and Protandrenini, and the addition of these would not be likely to alter biogeographic reconstruction.

Ancestral distributions within the family Melittidae are reconstructed with reasonable confidence. Melittidae has its greatest genetic, tribal, and subfamily diversity in Africa, and is reconstructed unambiguously as African in origin, as are its subfamilies, Melittinae and Dasypodainae (the third subfamily, Meganomiinae, is entirely restricted to Africa).

Andrenidae is reconstructed with weak support as primitively New World, consistent with the observation that basal genera of Andreninae, all Oxaeinae, and many Panurginae are restricted to the Americas [41,42]. For Halictidae, our results also weakly support a New World origin. Within Halictidae, lineages with a mix of both New and Old World taxa (e.g., Rophitinae, Nomiinae, and Halictinae) are reconstructed in our analysis to be New World in origin, and the monophyletic groups Halictinae + Nomioidinae + Nomiinae and Halictinae + Nomioidinae are weakly supported as ancestrally New World. This is in contrast to a previous analysis that hypothesized these lineages had origins in Africa, based on a flawed assumption that Nomiinae was likely of African origin [43].

The common ancestor of Stenotritidae and Colletidae is weakly supported as South American, with the split between ancestrally South American Colletidae and Australian Stenotritidae suggesting an ancient vicariance between South America and Australia [34]. Consistent with Almeida et al. [34], we find evidence of multiple interchanges between South America and Australia, presumably via Antarctica, over the course of colletid evolution. The colletid subfamilies Euryglossinae (+ Scrapterinae) and Hylaeinae are reconstructed as unambiguously Indoaustralian (Oceania) and the subfamily Xeromelissinae is reconstructed as unambiguously South American. Scrapterinae, the sole endemic African subfamily, appears to have arisen (via dispersal) from the Indoaustralian Euryglossinae (as hypothesized in [34]).

The ancestral state for Apidae as a whole is not clearly resolved. However, certain groups show clear connections with the New World. The “cleptoparasitic clade” of Apidae [32] is unambiguously reconstructed as South American. The corbiculate clade, as well as the monophyletic group including corbiculates and Centridini, are reconstructed as ancestrally New World. Xylocopinae, a widespread group, is entirely ambiguous. This could be due to the fact that three of the four xylocopine subfamilies (Ceratinini, Allodapini, and Xylocopini) are stem- or wood-nesters and dispersal over water appears to be fairly common in such bees [3,41,44,45]. Large bees, such as Xylocopini, may also be capable of long-distance dispersal via flight. Our results for Anthophorini are unclear because of limited taxon sampling and because this group is geographically widespread. For a more detailed treatment of anthophorine historical biogeography, see Dubitzky [46].

For Megachilidae, our results largely support the hypotheses proposed by Litman et al. [33]. Fideliinae, a paraphyletic group at the base of Megachilidae, has one lineage in South America (*Neofidelia*) and one in Africa (*Fidelia*), consistent with an ancient vicariance event between South America and Africa. The uncertainty in the ancestral reconstruction of Lithurginae is not unexpected, as bees in this group are widely-distributed and wood-nesting. Bees that nest in wood or preexisting cavities have a disproportionally high probability of long distance, human-mediated dispersal, and they may also be capable of dispersing over water via rafting [44,45]. Wood- and cavity-nesting bees are among the most common introduced bee species in North America. Of the 21 species of bees accidentally or intentionally introduced into North America, 14 are in the family Megachilidae [45]. Of the 17 exotic bee species reported in Canada, ten are in the family Megachilidae [47].

Our results would generally support a southern hemisphere origin for bees, because at the highest levels ancestral state reconstructions indicate strong connections among South America, Australasia (Oceania), and Africa. The split between Melittidae (Africa) and the remaining bee groups, many of which have inferred origins in the New World (especially South America), is consistent with the hypothesis that Gondwanan fragmentation impacted early bee evolution, as has also been suggested for Megachilidae [33]. Our results are consistent with a hypothesis, proposed by Michener [3,41], that bees arose in the xeric interior of Gondwana, particularly West Gondwana (Africa-South America).

### Future directions

Diversification among bee species has implications for processes driving early angiosperm diversification (e.g., [48]), but branching patterns early in bee history still remain unresolved. Statistical support for diversification patterns could be improved by increasing the number of genes sampled, and thus the number of characters that may be informative about that diversification. Sampling could be appropriately increased either by sequencing additional species for some of the more poorly-sampled genes (for example, abdA, ak, cad, and ecrb1 have all been successfully sequenced across Apoidea; Table 1), by using general arthropod primers to increase the number of genes sampled [29], or by utilizing large-scale sequencing strategies such as transcriptomics (e.g., [25,49]) or targeted enrichment (using sequenced bee genomes, as in [50-53]).

Given the comparatively poor sampling of the bees’ closest evolutionary relatives, the root of the bee tree and thus early diversification patterns of bees, may be resolved by increasing the taxonomic and gene sampling of apoid wasps. To improve reconstruction of the early geography of bee diversification, further data would also need to be collected on biogeographical ranges of these wasp taxa.

## Conclusions

Our study includes the largest number of bee genera for any study to date. We have reconstructed all families as monophyletic and can reject several proposed hypotheses for relationships among families. Our ability to reconstruct biogeographic patterns in bees at the highest levels indicates the utility of the supermatrix approach for historical biogeographic analysis. By including a much broader taxonomic and geographic sample of bees than has been included in previous studies of family-level relationships (e.g., [12]), we can more accurately reconstruct ancestral states using model-based methods. Supermatrix methods, and the insights derived from analysis of the massive amount of sequence data currently publically available, are therefore a powerful approach for inferring patterns on a broad evolutionary scale.

## Methods

### Sequence collection and alignment

All nuclear, coding DNA sequences for apoid wasps and bees were downloaded from the non-redundant nucleotide database of GenBank in October, 2011, and parsed using a custom Perl script. Of these, we only retained coding regions for those twenty genes that were represented by at least three bee tribes (Table 1): abdominal A (abdA), arginine kinase (ak), mitotic checkpoint control protein (bub3), calcium/calmodulin-dependent protein kinase II (cad), carbamoylphosphate synthetase/aspartate transarbamylase/dihydroorotase (camkii), deoxyribonucleoside kinase (dnk), ecdysone receptor B1 (ecr-b1), elongation factor 1-α f1 and f2 copies (ef1af1, ef1af2), feminizer (fem), glycerol kinase (gk), sodium potassium adenosine triphosphate (nak), odorant receptor 2 (or2), phosphoenolpyruvate carboxykinase (pepck), long wavelength rhodopsin (rho), RNA polymerase II (polII), ultraspiracle (usp), vasa (vas), white, and wingless (wg)*.* Because whole genomes are not available for most bee species, we cannot be certain that all of the nucleotide sequences identified for a given gene represent orthologs. However, paralogous copies were not identified after performing a search for each gene against the *Apis mellifera* genome [54] using blastn [55], and many of these genes are standard in bee phylogenetic analyses because they appear to be single-copy [8]. Under the assumption that members of a species are monophyletic, we selected the longest available sequence per species—longer sequences potentially contain more phylogenetically-informative characters—or one at random if there were more than one equally-long sequence for a particular species. These nucleotide sequences were aligned using MUSCLE v. 3.8 [56]. Minor adjustments were made by hand using Mesquite v. 2.73 [57] to retain amino acid coding and to remove introns in those records where they had not been annotated. This initial data set included 1666 species (summarized in Table 1; GenBank accession numbers in Additional file 5).

We removed any species represented by only one gene using a custom Perl script; thus each data partition contains overlap with at least one other partition for each taxon in our dataset. 1376 species remain in the alignment, spanning 374 genera, with a total alignment length of 22,612 sites (summarized in Table 1). After trimming the ends of each gene to remove sites with less than three taxa, 17,269 sites remain (alignment deposited in TreeBase under study accesstion number S14049). 85% of this matrix contains missing data (empty cells; distribution in Additional file 2). For generic-level analyses, we generated ten replicate alignments by randomly selecting one DNA sequence per gene per genus, removing genera represented by only one gene. Thus, each genus could be chimeric, containing sequences from different species. The average proportion of missing data across these ten generic alignments was reduced to 77%. 376 genera remain: 349 bee genera (~67% of genera [31]) and 27 apoid wasp outgroups. Finally, we also produced species-level alignments with more stringent rubrics for gene inclusion: one requiring genes to be sampled for two or more bee families (10 genes, 1336 taxa, 11944 sites, 78.4% missing), and one requiring genes to be sampled for at least four bee families (7 genes, 1328 taxa, 8467 sites, 71.7% missing).

### Phylogenetic analyses

We used jModelTest v.0.1 [58] to find the best-fit model of sequence evolution for each partition separately, and found the maximum-likelihood estimate under that model using Garli v.2.0 [59] with 20 search replicates. Nonparametric bootstrapping was performed with 2 search replicates per 100 bootstrap replicates.

Maximum-likelihood estimates for our concatenated alignments were generated using RAx-ML v. 7.2.8-alpha [60]. For our species-level and one randomly-selected generic alignment, we ran analyses under six partitioning schemes with the GTR-CAT approximation for sequence evolution: unpartitioned, partitioned by gene, partitioned by codon position, partitioned by codon positions 1+2 and 3, partitioned by 1+2 and 3 by gene, and partitioned by codon position within genes. We used the Akaike Information Criterion to determine the best-fit partitioning scheme. We ran 100 bootstrap replicates using this best-fit partitioning scheme, and used this pool of trees to calculate the taxon instability score using Mesquite [57]. We removed those taxa with instability scores in the top 1% from each alignment (n = 14 for species-level; n = 4 for generic-level), and re-ran analyses to find the maximum-likelihood estimate and bootstrap proportions. For our species-level and one randomly-selected generic alignment, we ran twenty replicate RAx-ML analyses to find the optimal maximum-likelihood estimate with 100 bootstrap replicates. In distantly-related or rapidly-evolving taxa, the third codon position can become saturated and potentially lead to inaccurate phylogenetic results due to the inability of the likelihood model to detect multiple substitutions. We ran an analysis in which the third codon position was excluded. The APE package in R [61] was used to plot bootstrap support on the species-level phylogeny. Figtree v.1.3.1 [62] was used to annotate clades at the tribal and subfamily levels using the tree from the generic-level analyses.

### Hypothesis testing

For each of eight alternative topological hypotheses (Figure 1), we constrained RAx-ML to find the best tree and site-likelihood scores under the GTRGAMMA model of sequence evolution using the species-level concatenated alignment. Constraints tested were: (1) Colletidae sister to the remainder of the bees (Figure 1H); (2) Colletidae + Stenotritidae sister to the remainder of the bees (Figure 1I); (3) Reciprocal monophyly of short-tongued and long-tongued bees (Figure 1J); (4) Andrenidae sister to Colletidae + Stenotritidae + Halictidae (Figure 1K); (5) Melittidae + Andrenidae sister to the remainder of the bees (Figure 1L); (6) Melittidae sister to all other bees, and Andrenidae sister to the remaining bees (Figure 1M); (7) Melittidae and long-tongued bees as a clade (Figure 1N); (8) Melittidae paraphyletic, with Dasypodainae sister to the remainder of the bees (Figure 1O). The log likelihood of any given tree is a sum of the log likelihood for each site. One method of examining whether one tree has a statistically significantly higher likelihood than another is to use the site likelihoods for each hypothesis. We generated 10,000 bootstrap replicates of the site likelihoods for each constrained tree using CONSEL [63], and ranked alternative hypotheses using the weighted Shimodaira-Hasegawa test (WSH [63]), the approximately unbiased test (AU [64]), and the Bayesian Information Critierion approximation for posterior probability (BIC [63]). These tests generate p-values indicating whether a topology can be rejected from the confidence set, and were selected as they range in power and sensitivity. Both the AU and WSH tests reduce selection bias inherent in comparing the maximum-likelihood estimate to less-likely trees. The AU test tends to work well when selection bias is not extreme, but is less conservative than the WSH as the true tree can be excluded from the confidence set when many of the best trees are nearly as good [64]. The more conservative WSH test tends to overestimate selection bias [64], and thus the number of trees in the confidence set increases with the number of trees being compared [65]. As we performed multiple statistical tests, we used a Bonferroni correction on the p-values [66] used for excluding a particular tree from the confidence set.

### Phylogenetic decisiveness

Not all possible taxonomic triplets in the concatenated data set are represented in individual gene alignments, which means that our taxon sampling is not phylogenetically decisive [36]. We calculated partial tree-wise phylogenetic decisiveness based on simulations of 1000 equiprobable, random trees [36] using the program decisivatoR [http://cores.ibest.uidaho.edu/software/decisivator] for our species-level, genus-level, and subsampled data sets. DecisivatoR required more than 24GB RAM to run our DNA sequence alignments, so we simplified our matrices to gene presence (1) or absence (?). We additionally estimated the number of trees that could be built from triplets in our data (i.e., tree terrace size [37]), and used these trees to calculate a ‘BUILD’ tree [38] using the Perl scripts in the package PhyloTerraces [http://sourceforge.net/projects/phyloterraces/].

### Biogeography

The current distributions of bee genera were assigned to one or more of seven broad geographic regions: Africa, Eastern Palearctic, Western Palearctic, North America, South America, Central America, and Oceania (i.e., the Australasian or Indoaustralian ecozone) (from [3]; Additional file 6). To calculate the posterior probability of ancestral distribution at internal nodes, we used a Bayesian approach implemented in the updated version of Statistical Dispersal Vicariance Analysis (S-DIVA), RASP v.2.0b [67,68], and our generic-level phylogeny (Figure 3). The wasp outgroups, which are relatively poorly sampled compared to the ingroup taxa and are biased towards North American taxa, were set to have a null distribution according the recommendation of the program author (Y. Yu, pers. comm.). The program was run for 1 million cycles along 10 chains, with a maximum number of areas occupied by a single taxon of 4. The state frequencies were set to the F81 model [69] with a gamma distribution for among-site rate variation. Default settings were used for all other program parameters. Parsimony and maximum-likelihood reconstructions were performed in Mesquite [57], with each geographic region scored as a separate, binary character (Additional file 6).

## Competing interest

The authors declare that they have no competing interest.

## Authors’ contributions

SMH designed the study, performed analyses, and drafted the paper. SP designed the study and performed analyses. BND designed the study and drafted the paper. All authors read and approved the final manuscript.

## Supplementary Material

Additional file 1 **Maximum-likelihood estimate of apoid species phylogenies.** Phylogenetic estimates of relationships among species based on maximum-likelihood analyses of DNA sequence data, in Newick format. Bootstrap support is based on 100 replicates. Click here for file

Additional file 2 **Distribution of missing data.** Genes used in phylogenetic estimation are labelled by site in the concatenated alignment; cut-offs for subsampled alignments (10-gene and 7-gene) are indicated. Click here for file

Additional file 3 **Maximum-likelihood phylogenetic estimates of apoid genera.** Ten replicate alignments were generated by randomly sampling one sequence per genus per gene. Bootstrap support is based on 100 replicates, after removal of ‘rogue’ taxa. Click here for file

Additional file 4 **Parsimony- and maximum-likelihood based reconstruction of bee historical biogeography.** Present geographic range was used in maximum-likelihood (ML) and maximum-parsimony (MP) framework to estimate ancestral presence or absence of a genus in each of 7 biogeographic areas. Click here for file

Additional file 5 **GenBank numbers for sequences used in this study.** Table with GenBank numbers for taxa and genes sampled. Click here for file

Additional file 6 **Geographic distributions of bee genera.** Presence/absence of each genus in each of seven geographic regions indicated with a 0 (absent) or 1 (present). Data from reference [3]. Click here for file
