# Supplementary figures and images for: The bee tree of life: a supermatrix approach to apoid phylogeny and biogeography
Source: BMC Evol Biol. 2013 Jul 3;13:138. doi: 10.1186/1471-2148-13-138 (PMC3706286; doi:10.1186/1471-2148-13-138)

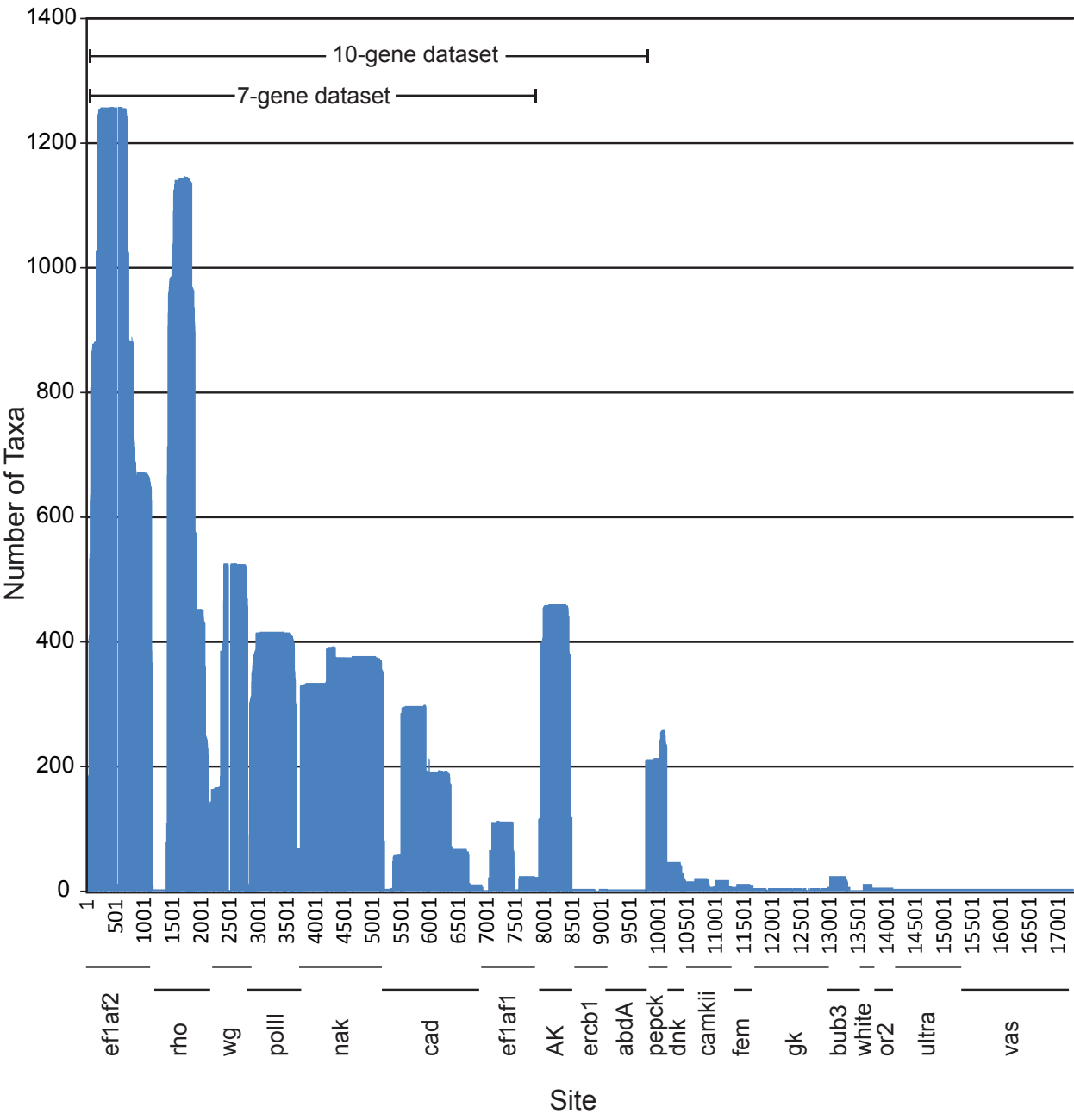

Supplement: Additional file 2 — Distribution of missing data. Genes used in phylogenetic estimation are labelled by site in the concatenated alignment; cut-offs for subsampled alignments (10-gene and 7-gene) are indicated. [file 1471-2148-13-138-S2.pdf]

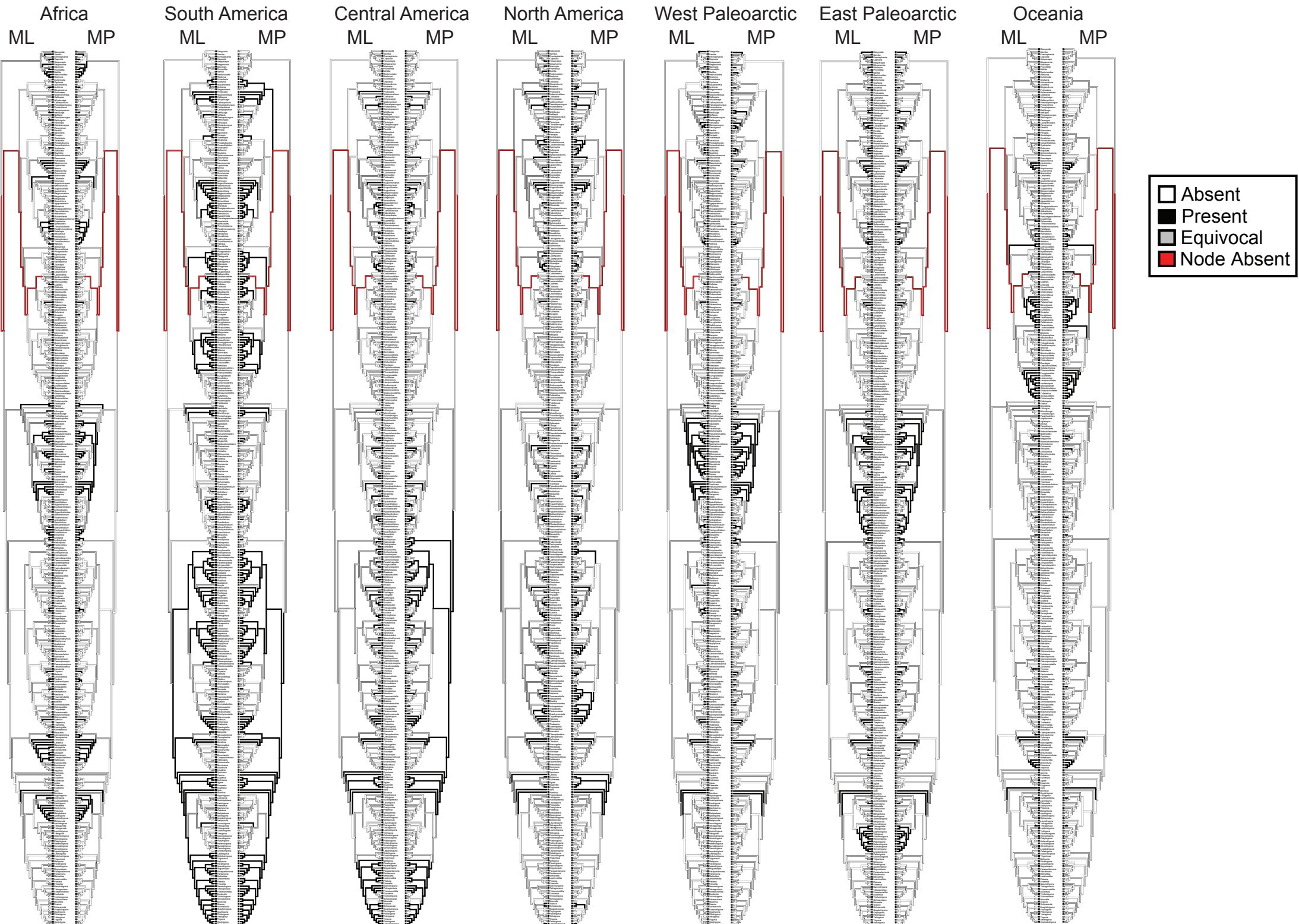

Supplement: Additional file 4 — Parsimony- and maximum-likelihood based reconstruction of bee historical biogeography. Present geographic range was used in maximum-likelihood (ML) and maximum-parsimony (MP) framework to estimate ancestral presence or absence of a genus in each of 7 biogeographic areas. [file 1471-2148-13-138-S4.pdf]
